# MicroRNA-214-3p Delivered by Bone Marrow Mesenchymal Stem Cells-Secreted Exosomes Affects Oxidative Stress in Alzheimer’s Disease Rats by Targeting CD151

**DOI:** 10.1080/15476278.2025.2489673

**Published:** 2025-04-27

**Authors:** Luzy Zhang

**Affiliations:** School of Pharmacy and Food Science, Zhuhai College of Science and Technology (Zhuhai College of Jilin University), Zhuhai, Guangdong, China

**Keywords:** Alzheimer’s disease, apoptosis, bone marrow mesenchymal stem cells, CD151, exosomes, microRNA-214-3p, oxidative stress injury

## Abstract

**Objective:**

This study probed the effect of targeted regulation of CD151 by microRNA-214-3p (miR-214-3p) delivered by bone marrow mesenchymal stem cells-secreted exosomes (BMSCs-exo) on oxidative stress and apoptosis of neurons in Alzheimer’s disease (AD).

**Methods:**

Rat BMSCs were isolated, from which MSCs-exo were extracted and identified. The AD rat model was established and injected with MSC-exo suspension. Meanwhile, miR-214-3p and CD151 interfering lentivirus were transfected in MSCs. After injection, learning and cognitive ability of the rats were assessed, as well as neuronal apoptosis and oxidative stress injury. miR-214-3p and CD151 levels were determined, and their relationship was explored.

**Results:**

AD rats had prolonged escape latency, weakened learning and cognitive ability, increased neuronal apoptosis in the hippocampal CA3 region, and aggravated oxidative stress. After MSC-exo injection, these changes in AD rats were partially rescued. CD151 was targeted by miR-214-3p, and MSC-exo improved AD in rats through the miR-214-3p/CD151 axis.

**Conclusion:**

MSC-exo down-regulates CD151 by targeting miR-214-3p to enhance antioxidant capacity, thereby improving the pathological injury of AD rats.

## Introduction

Alzheimer’s disease (AD) accounts for 50–75% of dementia cases, and the prevalence doubles roughly every five years after age 65.^1^ The early stages of the disease are characterized by difficulties in encoding and storing new memories. Subsequent progressive cognitive and behavioral changes accompany the later stages.^2^ Accumulated oxidative stress has been viewed as a key mechanism leading to AD.^3^ The brain is more susceptible to oxidative stress than other organs, and most of the neuronal components can be oxidized. Oxidative stress is involved in AD by promoting tau hyperphosphorylation, amyloid-β (Aβ) deposition, and synapse and neuron loss.^4^ In view of this, targeting oxidative stress has therapeutic potential in the prevention and management of AD.

Exosomes (exos) are small vesicles outside the cell that carry biologically active substances. In the brain, exo may be derived from almost all types of cells and is involved in cell communication to regulate cell function.^5^ By carrying substances such as lipids, proteins, DNA, miRNAs, mRNAs, and lncRNAs, exos perform crucial roles in maintaining cellular homeostasis, clearing cellular waste, and facilitating intercellular and inter-organ communication. These exos can freely move through various bodily fluids and transmit their molecular cargo through paracrine, autocrine, and endocrine mechanisms.^6^ Mesenchymal stem cells-derived exosomes (MSCs-exos) are emerging alternatives for AD treatment due to their donor-derived properties, minimal immunogenicity, ease of storage, natural delivery vector, and low risk of tumor formation.^7^ It has been reported that MSCs-exos improve cognitive function and increase subventricular zone neurogenesis in AD mice.^8^ The fact that exos can cross the blood-brain barrier enhances the promising therapeutic potential of exos in AD. Therefore, exos can be suitable candidates for gene therapy because of their ability to deliver nucleic acids specifically.^9^ Primitive MSCs can be obtained from various sources, including bone marrow, adipose tissue, and umbilical cord blood.^10^ It is noteworthy that bone marrow mesenchymal stem cells (BMSCs) are considered the most efficient exosome-producing cells.^11^ BMSCs are prototypical stem cells capable of differentiating into various cell types under different physiological conditions. They can selectively migrate to sites of tissue injury, interact with brain cells, and subsequently stimulate the production of growth factors such as brain-derived neurotrophic factor and nerve growth factor. Most importantly, BMSC exosomes exhibit similar effects to BMSCs but without adverse side effects.^11^ Yalan Lu et al. have indicated that the secretory factors IGF1, VEGF, and Periostin2 derived from BMMSCs play a crucial role in neuroprotection by restoring neuronal function through modulation of the AKT/IAPs pathway. These cytokine sets may represent potential strategies for the treatment of attention deficit disorders.^12^

microRNAs (miRs) perform biological functions by regulating intracellular protein expression and are found in different abundance and species in the central and peripheral tissues of AD patients and healthy populations.^13^ miR-214-3p is a novel potential neuroprotective factor for AD.^14^ Several reports have confirmed that miR-214-3p is associated with neuronal autophagy,^15^ proliferation, and apoptosis.^16^ miRNAs, through their actions, shape the transcriptome of brain cells and are generally associated with very high genetic activity, gene transcription, and the production of mRNA.^17^ CD151 was predicted as a target of miR-214-3p in this study. Tetraspanins are a family of universally expressed and conserved proteins that can regulate the proteolysis of amyloid precursor protein.^18^ To our best knowledge, tetraspanin CD151 has not been discussed in AD.

Therefore, this study evaluated the treatment potential of miR-214-3p delivered by MSCs-exo in AD through targeting CD151, aiming to provide a novel reference for drug delivery systems and miRNA-targeted therapy for AD.

## Materials and methods

### Ethics statement

Experiments in this study were strictly followed by the guidelines and procedures approved by the Institutional Use and Care Committee of our Technology. Every effort was made to minimize the suffering of animals.

### Extraction and identification of rat BMSCs

To isolate bone marrow, Sprague Dawley rats were anesthetized via intraperitoneal injection of sodium pentobarbital (50 mg/kg). After sufficient anesthesia, cervical dislocation euthanasia was performed, and the hind limbs were dissected from the trunk, with care taken to avoid damaging the femur. The knee joint was cut, the muscles and connective tissue were removed from the tibia and femur, and the tibia and femur ends were incised. Bone marrow was rinsed with Dulbecco’s Modified Eagle’s Medium (DMEM, Gibco, USA) using a 27-gauge needle attached to a 10 ml syringe. After centrifugation, cell precipitations were re-suspended in 15% fetal bovine serum (FBS, Gibco)-DMEM with 5% CO_2_ at 37°C for 48 h. MSCs were isolated with 0.25% trypsin ethylenediamine tetraacetic acid (Gibco-Invitrogen, USA) and subcultured in 15% FBS-DMEM. Flow cytometry was adopted to analyze the expression of surface markers on MSCs. MSCs in logarithmic growth phase were washed twice with phosphate buffered saline (PBS) and adjusted to a concentration of 1 × 10^6^ cells/mL. Then, 100 μL of cell suspension was added with 5 μL of fluorescently conjugated antibodies, including CD29-FITC (12-0291-82, Thermo), CD44-PE (14-0441-82, Thermo), CD31-APC (11-0311-82, Thermo), and CD45-PE-Cy7 (14-0451-82, Thermo). The cells were incubated in the dark for 30 minutes at 4°C, washed twice with PBS, and centrifuged at 400 g for 5 min. The supernatant was discarded, and the cells were resuspended in 300 μL of PBS. Detection was performed using a FACSCanto^TM^ II flow cytometer (Becton Dickinson, Franklin Lakes, NJ, USA), and data were analyzed using FlowJo v10.8.1 software. The proportion of positive cells was set based on IgG isotype controls. MSCs highly expressed CD29 and CD44 and lowly expressed CD31 and CD45, distinguishing them as derived from mesenchymal stem cells rather than hematopoietic or endothelial cells.^19^

The multidirectional differentiation capacity of MSCs was evaluated through osteogenic and adipogenic induction experiments. Cells were seeded in 6-well plates and cultured until 80% confluence, then induced with osteogenic induction medium (Cyagen, China) and adipogenic induction medium (Cyagen, China) for 14 days, with medium changes every 3 days. Osteogenic induction was assessed using Alizarin Red S staining to detect mineralized nodule formation, and adipogenic induction was evaluated using Oil Red O staining to detect lipid droplet formation.

### Isolation and identification of MSCs-exo

MSCs were centrifuged at 200,000 × g for 18 h to remove exo from the medium and cultured in an exo-free medium containing 10% FBS. MSCs at passages 4–6 were collected for exo collection. MSCs (2 × 10^6^) were placed in a 100 mm petri dish for 72 h, and 10 mL supernatant was taken, centrifuged at 300 × g (10 min), 1200 × g (20 min), and 10,000 × g (30 min) at 4°C, and then filtered through a 0.22 μm filter (Millipore, MA, USA). The filtrate was centrifuged in an L-80XP ultra-centrifuge (Beckman Coulter, CA, USA) at 140,000 × g (90 min) at 4°C, and exo was re-suspended with PBS and centrifuged again at 140,000 × g (90 min). After that, exo was re-suspended in PBS and tested by a bicinchoninic acid (BCA) kit (Thermo Fisher Scientific).

Transmission Electron Microscopy (TEM) was performed. A 20 μL aliquot of freshly ultracentrifuged exosome samples was fixed with 1% glutaraldehyde, loaded onto a carbon-coated copper electron microscope grid for 2 min, and negatively stained with a 1% phosphotungstic acid solution (12501-23-4, Sigma-Aldrich, USA) for 5 min. The samples were dried under an incandescent lamp and photographed using a JEM-2100 TEM (JEOL, Tokyo, Japan).^20^

Additionally, Western blot analysis was performed to detect the expression of exosome-specific surface markers CD9, CD81, TSG101, and the non-exosome marker CANX.

### AD model establishment and animal treatment

Sixty-four Sprague-Dawley male rats (12 months old, 290–360 g) were adapted to the environment and had free access to water and feed one week before the experiment. The rats were kept at 22–24°C, humidity of 50–60%, and 12-h light/dark cycles. The rats were randomly divided into 8 groups, 8 in each group, including sham, model, MSCs-exo, MSCs-exo + anti-miR-NC, MSCs-exo + anti-miR-214-3p, MSCs-exo + anti-miR-NC + sh-NC, MSCs-exo + anti-miR-214-3p + sh-NC, and MSCs-exo + anti-miR-214-3p + sh-CD151 groups. In the model group, rats received 30 mg/kg of pentobarbital sodium intraperitoneally (Jiangsu Hengrui Pharmaceuticals Co., Ltd., Jiangsu, China). Aβ_1–42_ (AnaSpec, CA, USA) was dissolved in sterile saline at 37°C for 48 h. After ether, 7 μg Aβ_1–42_ was slowly injected into the right ventricle for 3 days. An equal volume of 0.9% sterile saline was injected into rats in the sham group. The exo suspension (100 μL) was injected intravenously within 5 h after modeling for 5 days.^19,21^ miR-214-3p low expression sequences, CD151 lentiviral vector, and NCs were acquired in GenePharma (Shanghai, China). BMSCs were cultured overnight in an antibiotics-free medium and lentiviral transfection (1.0 × 10^9^ TU/ml) was performed at 30–50% confluence.^22^

### Morris water maze (MWM)

Two weeks after injection, MWM was implemented with a transparent platform, a circular pool (120 cm × 50 cm × 30 cm), and an automatic recording system. A room-temperature water supply was maintained. The platform was placed in the first quadrant, and two entry points were selected on the two opposite sides of the platform that are the same distance from the platform. Rats were adapted to the maze 1 day earlier. The platform was fixed during testing. The rats faced the pool wall and slowly put into the water from 3 points (except the entry point in the fourth quadrant). The escape latency was recorded, and the rats stayed on the platform for 10 s. If the rat did not find the platform within 60 s, the escape latency was recorded as 60 s and the rat was trained at the next entry point. When the rats entered the pool from 3 points respectively, the average latency was regarded as the escape latency. Based on the latency, spatial learning and memory abilities were determined. As the latency decreases, spatial learning and memory improve. Experiments were performed on all rats prior to modeling. After 3 days of training, the latency was recorded twice a day for 5 days. The escape latency is taken from the daily average latency.^23,24^

### Tissue sampling

After MWM, the rats were anesthetized with 30 mg/kg pentobarbital sodium. After heart infusion, the whole brain was removed, and the CA3 region was cut into 1 × 1 × 1 mm^3^ blocks and frozen at −80°C. Then, the blocks were processed with fractional ethanol dehydration and clearance in xylene I and II. Next, the blocks were immersed in paraffin (I, II, and III) at 50°C and then placed in paraffin IV. Paraffin blocks were sectioned at 4–8 μm and air-dried for 2 h for tissue staining and real-time quantitative polymerase chain reaction (RT-qPCR) and enzyme-linked immunosorbent assay (ELISA).

### TUNEL staining

The sections were stained, rinsed with xylene, gradient ethanol, and PBS, added with protease K, and rinsed with distilled water according to the operating instructions of TUNEL kit, and apoptosis was observed under optical microscopy. Those with brown-yellow granules in the nucleus were positive cells, namely apoptotic cells. Ten fields were randomly selected in each section and the total cell number and apoptotic cell number were recorded. TUNEL positive rate = TUNEL-positive cell number/total cell number × 100%.

### Detection of glutathione peroxide (GSH-Px), superoxide dismutase (SOD), reactive oxygen species (ROS), and malondialdehyde (MDA)

Hippocampal CA3 tissue homogenate was prepared. SOD was determined by the xanthine oxidase method, GSH‐Px activity and ROS by the Fenton method, and MDA by the thiobarbituric acid method. SOD kit (A001), GSH‐PX kit (A005), LDH kit (A020–2), ROS kit (A003–2), and MDA kit (A003–1) were provided by Nanjing Jiancheng (Jiangsu, China).^23^

### RT-qPCR

Total RNA was extracted by Trizol (Invitrogen, CA, USA). miRNA and mRNA cDNAs were produced using Mir-X miR First-Strand Synthesis kit (Takara, Dalian, China) and PrimeScript RT reagent Kit (Takara), respectively, followed by detection by SYBR Premix Ex Taq II (Takara). PCR was conducted in the ABI Prism 7500 Fast Real-Time PCR system (Applied Biosystems, MA, USA). PCR conditions were set: denature at 95°C for 10 min and 40 cycles at 94°C for 30 s, 59°C for 30 s, and 72°C for 30 s. Gene expression was calculated by the 2^−ΔΔCt^ method with U6 and β-actin as internal references respectively. Primer sequences are shown in Supplementary Table 1.

### Western blot

The tissue was placed in a centrifugation tube, and 100 μL radio-immunoprecipitation assay lysis buffer (Solarbio, Beijing, China) containing 1 mmol/L phenylmethylsulfonyl fluoride was added and homogenized at 3000 r/min. Proteins were placed on ice for 30 min and centrifuged at 4°C at 12,000 × g for 4 min. The supernatant was harvested and stored at −80°C. The protein concentration was determined by the BCA kit (Boster, Hubei, China). The extracted protein (3 μg/μL) was added to the sample buffer and boiled for 10 min at 95°C. The sample was 30 μg per well and the protein was isolated by 10% polyacrylamide gel electrophoresis, transferred to polyvinylidene difluoride membranes (Sigma, USA) by semi-dry method, and blocked with 5% bovine serum albumin (Beijing Zhongsheng Likang Technology Co., Ltd., China) for 1 h, and rabbit CD9 (ab236630, 1:1000), CD81 (ab219209, 1:1000), TSG101 (ab125011, 1:1000), CANX (ab22595, 1:1000), and β-actin (ab8226, 1:10000, all from Abcam) were added overnight at 4°C. The membranes were added with the corresponding sheep anti-rabbit secondary antibody (ab6721, Abcam, 1:2000) for 1 h and developed by enhanced chemiluminescent reagent in the Bio-rad Gel Doc EZ IMAGER (Bio-rad, USA). Gray values were assessed by Image J software.

### Luciferase activity

Target gene analysis of CD151 3’UTR with miR-214-3p was conducted on the website TargetScan (http://www.targetscan.org/vert_71/). CD151 luciferase reporter and mutant reporter were constructed: pGLO-CD151 wild type (Wt) and pGLO-CD151 mutant type (Mut). The two reporters were co-transfected into HEK293 cells with miR-214-3p mimic and pRL-TK. At 48 h post-treatment, the Dual-Luciferase Reporter Assay System (E1910; Promega, Madison, WI, USA) was used, and luciferase activity was measured using a SpectraMax iD5 Multi-Mode Microplate Reader (Molecular Devices, San Jose, CA, USA).

### Statistical analysis

All data were processed by SPSS 21.0 (IBM, IL, USA). Measurement data conforming to normal distribution and homogeneity of variance were represented by mean ± standard deviation. The independent sample t-test was utilized to compare the two groups of unpaired data. Data among multiple groups were comparatively assessed by one-way analysis of variance (ANOVA) and Tukey’s post hoc test. It was statistically significant that the difference was *p* < 0.05.

## Results

### Isolation and identification of BMSCs and MSCs-exo

**Figure 1.**
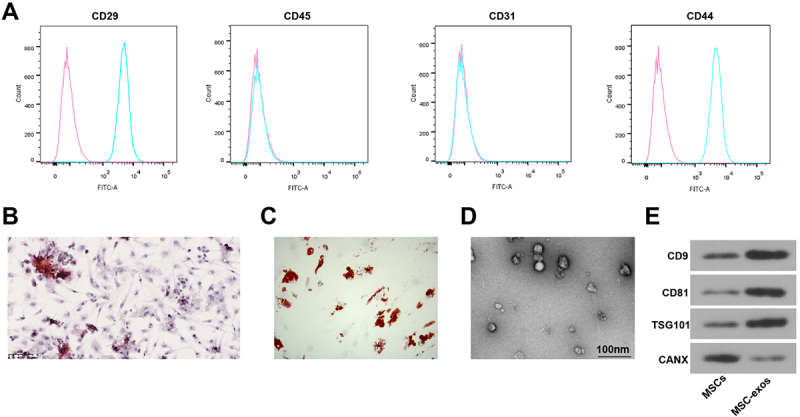
Isolation and identification of bone MSCs and MSCs-exo. (A) flow cytometry analysis of MSCs-related surface markers, with pink representing the isotype control and cyan representing the specific marker; (B) Alizarin red staining detection of osteogenic differentiation of MSCs; (C) oil red O staining detection of adipogenic differentiation of MSCs; (D) TEM observation of exos; E: Western blot detection of exo-specific surface proteins. N = 3; each experiment was repeated three times.

MSCs were isolated from rat bone marrow, and surface markers of MSCs were identified by flow cytometry. CD29 and CD44 were highly expressed in the isolated cells, while CD31 and CD45 were lowly expressed (Figure 1(A)). At the same time, multi-differentiation experiments found that the obtained cells had good potential for osteogenic and adipogenic differentiation (Figure 1(B-C)). Therefore, cells isolated from rat bone marrow were confirmed as MSCs.

Exo was isolated and characterized by differential centrifugation. TEM observation showed that MSCs-exo had circular vesicles with lipid bilayer membrane structure (Figure 1(D)). Western blot analysis also found that CD9, CD81, and TSG101 in the obtained exo were elevated, while CANX expression was absent (Figure 1(E)), indicating successful exo separation.

### MSCs-exo improves pathological injury in AD rats

**Figure 2.**
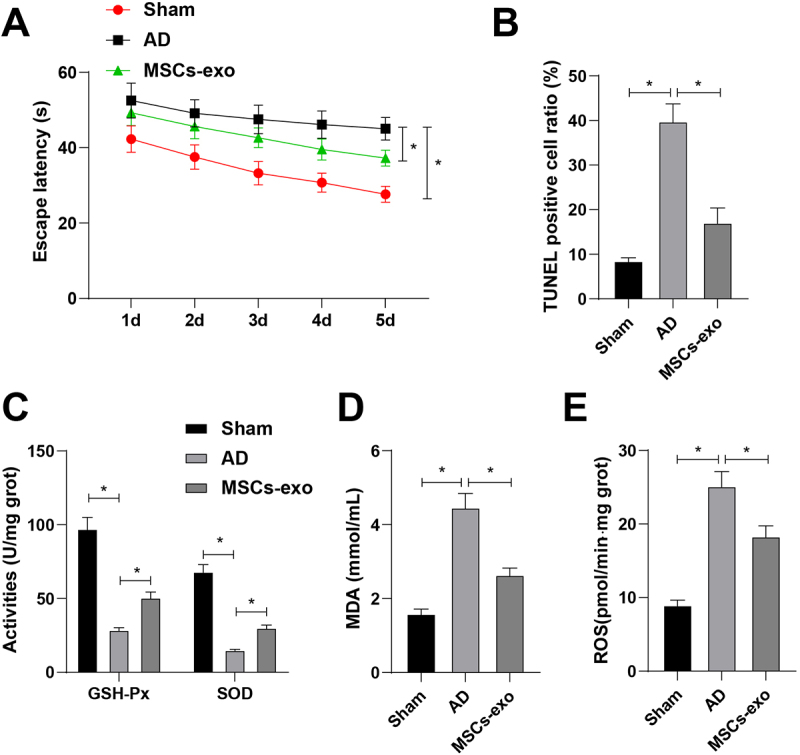
Effects of bone MSCs-exo on pathological damage in AD rats. (A) MWM detected behavioral activities of rats; (B) TUNEL staining detected neuronal apoptosis in the hippocampal CA3 region; (C) GSH-Px and SOD activity were detected in the hippocampal CA3 region of rat brains among different groups; (D) MDA levels were detected in the hippocampal CA3 region of rat brains among different groups; (E) ROS levels were detected in the hippocampal CA3 region of rat brains among different groups. The values in the figure are measurement data, presented as mean ± standard deviation. One-way ANOVA was used for comparison among multiple groups, followed by Tukey’s post-hoc test. *p < 0.05; N = 3; each experiment was repeated three times.

To further explore the effect of MSCs-exo on the behavior of AD rats, MWM was performed. The escape latency of rats was prolonged after AD modeling, while shortened by MSCs-exo injection (Figure 2(A)).

TUNEL staining evaluated neuronal apoptosis in the hippocampal CA3 region. Neuronal apoptosis was detected to be activated after AD modeling, and MSCs-exo treatment had an alleviating effect on it (Figure 2(B)).

Regarding oxidative stress injury, it was measured that AD modeling led to impairments in GSH-Px and SOD activities in the hippocampal CA3 region, together with elevated MDA and ROS levels. AD-induced oxidative stress injury was attenuated by MSCs-exo, as reflected by reduced MDA and ROS and elevated GSH-Px and SOD (Figure 2(C-E)). Overall, MSCs-exo improves pathological injury in AD rats.

### MSCs-exo influences AD through miR-214-3p

**Figure 3.**
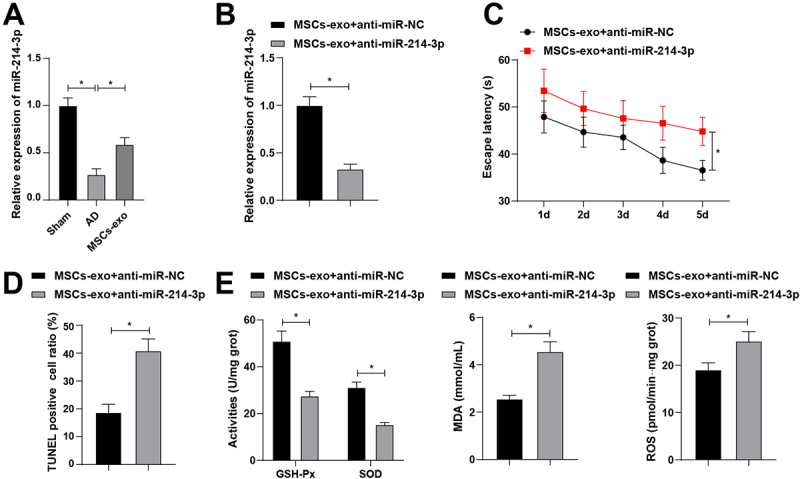
Bone MSCs-exo influences AD via miR-214-3p. (A-B) Rt-qPCR detection of miR-214-3p in the CA3 region of rat hippocampus; (C) MWM detected behavioral activities of rats; (D) TUNEL staining detected neuronal apoptosis in the hippocampal CA3 region; (E) oxidative stress in hippocampal CA3 region. The values in the figure represent measurement data and are presented as mean ± standard deviation. For comparisons between two groups, an independent-sample t-test was used. For comparisons among multiple groups, one-way ANOVA was employed, followed by Tukey’s post-hoc test. *p < 0.05; N = 3; each experiment was repeated three times.

RT-qPCR measured miR-214-3p low expression in the hippocampal CA3 region of AD rats and confirmed the upregulating effect of MSCs-exo injection on miR-214-3p expression (Figure 3(A)). Therefore, follow-up experiments explored miR-214-3p in improving AD. miR-214-3p low expression vector was successfully transferred into MSCs-exo, leading to reduction in miR-214-3p expression (Figure 3(B)). Consequently, it was observed that miR-214-3p downregulation in MSCs-exo prolonged the escape latency of rats (Figure 3(C)), enhanced neuronal apoptosis (Figure 3(D)), as well as impaired GSH-Px and SOD while forced MDA and ROS levels (Figure 3(E)). In short, MSCs-exo influences AD through miR-214-3p.

### miR-214-3p inhibits CD151 expression

**Figure 4.**
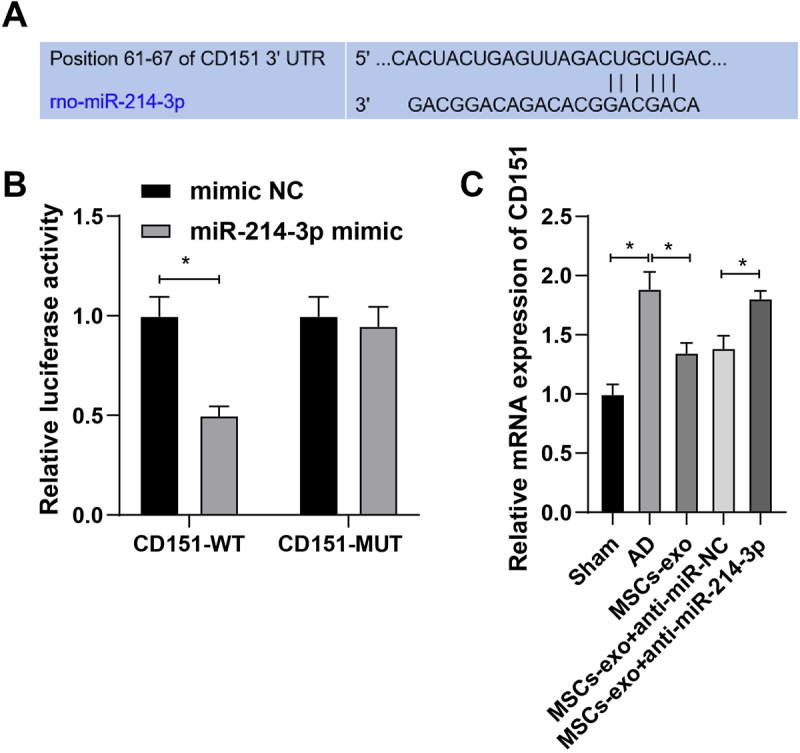
miR-214-3p inhibits CD151. (A) bioinformatics predicted the binding sites of miR-214-3p and CD151; (B) dual luciferase reporter gene assay verified the targeting relationship between miR-214-3p and CD151; (C) Rt-qPCR detection of CD151 in hippocampal C3 region. The values in the figure are measurement data, presented as mean ± standard deviation. One-way ANOVA was used for comparison among multiple groups, followed by Tukey’s post-hoc test. *P < 0.05; N = 8; each experiment was repeated three times.

Bioinformatics predicted the existence of binding sites between miR-214-3p and CD151 3’UTR (Figure 4(A)). Dual luciferase reporter gene assay verified the targeting relationship between miR-214-3p and CD151 (Figure 4(B)). Further RT-qPCR assessed CD151 in the hippocampal C3 region and detected higher CD151 expression in AD rats. CD151 expression decrease was detected when MSCs-exo were injected. However, the miR-214-3p low expression vector delivered by MSCs-exo restored CD151 expression (Figure 4(C)). It is suggested that miR-214-3p inhibits CD151 expression in AD rats.

### MSCs-exo improves AD by targeting CD151 via miR-214-3p

**Figure 5.**
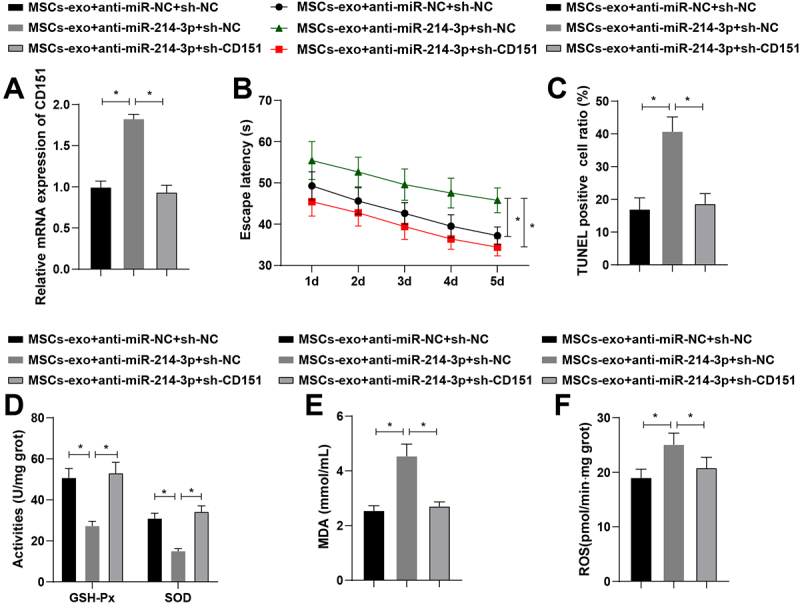
Bone MSCs-exo influences AD through the miR-214-3p/CD151 axis. A: RT-qPCR detection of CD151 in the hippocampal CA3 region; B: MWM detected behavioral activities of rats; C: TUNEL staining detected neuronal apoptosis in the hippocampal CA3 region; D: GSH-Px and SOD activity were detected in the hippocampal CA3 region of rat brains among different groups; E: MDA levels were detected in the hippocampal CA3 region of rat brains among different groups; F: ROS levels were detected in the hippocampal CA3 region of rat brains among different groups. The values in the figure represent measurement data and are presented as mean ± standard deviation. For comparisons between two groups, an independent-sample t-test was used. For comparisons among multiple groups, one-way ANOVA was employed, followed by Tukey’s post-hoc test. *P < 0.05; N = 3; each experiment was repeated three times.

CD151 interference vector was constructed and transfection efficiency was verified by RT-qPCR assay as shown by a reduction in CD151 expression (Figure 5(A)). Interfering with CD151 expression rescued the pro-AD impacts of MSCs-exo expressing low miR-214-3p, contributing to shortened escape latency (Figure 5(B)), reduced apoptosis (Figure 5(C)), and attenuated oxidative stress injury (Figure 5(D-F)). Shortly, MSCs-exo improves AD in rats through the miR-214-3p/CD151 axis.

## Discussion

As the world’s population ages, the prevalence of AD continues to grow.^25^ Current drug development efforts for AD have failed to produce effective disease modifiers for reasons, and alternative non-drug approaches to detect and treat early pathological events are urgently needed, along with new molecular targets, biomarkers, and diagnostic techniques.^26^ Studies have shown that MSCs, a group of pluripotent stem cells, can stimulate nerve regeneration and inhibit disease progression.^27^ Recently, exos derived from MSCs provide a novel perspective on therapeutic strategies of AD their immunomodulatory and neuroprotective effects, similar to those of the host human MSCs.^7^ In view of this, this study primarily probed the ability of MSCs-exo carrying miR-214-3p in alleviating oxidative stress injury of AD rats.

Alone, MSCs-exo alleviated pathological damage in AD rats by improving behavioral function, reducing neuronal apoptosis, and enhancing the anti-oxidant stress mechanism. Lateral ventricle administration of BMSC-exos in a mouse model of AD can improve behavioral function.^11^ Moreover, rabies viral glycoprotein-modified MSC-exo effectively target cortex and hippocampus and therefore improve learning and memory capabilities in AD mice.^19^ MSCs-exo stimulate neurogenesis in the subventricular zone and alleviate cognitive deficits.^8^ Moreover, a current paper illustrates the neuroprotective actions of MSCs-exo in AD mice by reducing Aβ deposition and improving cognitive function recovery.^28^ Also, intranasal administration of MSCs-exo reaches the hippocampus and neocortex of mice in the AD model and improves spatial and memory abilities.^29^ Sorts of studies have verified that MSCs-exo present an anti-oxidative stress mechanism in disease development, including cerebral ischemia-reperfusion injury,^30^ intracerebral hemorrhage,^31^ and Parkinson’s disease.^32^ Studies focus on the functionalization and engineering strategies (such as drug loading, and surface modification) of MSCs-exo, which can improve the treatment of AD by clearing abnormal protein accumulation and achieving multiple therapeutic effects such as neuroprotection and immune regulation.^33^ This study confirms that BMSC-exos have a positive effect on improving the pathological damage in AD rats.

Within exosomal cargoes, a wide range of miRNAs has been found that can control functions related to neuro-remodeling as well as angiogenesis and neurogenesis processes.^34,35^ The study focus lies on miR-214-3p loaded by MSCs-exo in AD. miR-214-3p was lowly expressed in the hippocampal CA3 region of AD rats, which was consistent with miR-214-3p downregulation in the plasma of AD patients.^16^ Functionally, suppressing miR-214-3p was determined to prevent behavioral function recovery and worsen neuronal apoptosis and oxidative stress injury. Generally, miR-214-3p provides a cognitive improvement effect on AD progression according to a brief review.^36^ Intriguingly, it is well displayed that downregulating miR-214-3p leads to neuronal autophagy and apoptosis.^14,15^ It is illustrative to report that miR-214-3p silencing promotes cognitive dysfunction and oxidative stress in rats induced by isoflurane.^37^ A close relationship exists between miR-214-3p and oxidative stress.^38,39^ This study found that when the miR-214-3p content in MSCs-exo was reduced, the escape latency of rats was prolonged, cellular apoptosis increased, and the state of oxidative stress worsened. This suggests that BMSCs-exo may improve AD symptoms by affecting the expression of miR-214-3p.

Furthermore, CD151 was validated to be a direct target of miR-214-3p in the process of AD. This study further analyzed CD151 expression patterns. RT-qPCR results showed higher CD151 expression in AD rats. Additionally, experiments validated that CD151 downregulation following miR-214-3p silencing protected AD rats. CD151 has attracted academic attention in the field of tumorigenesis and is widely defined as a tumor promoter.^40–42^ However, CD151 in AD processes needs further analysis and validation.

In brief, miR-214-3p delivered by BMSCs-exo relieves cognitive dysfunction, oxidative stress, and apoptosis in AD rats by negatively inhibiting CD151. This study was limited to animal models, and the function of MSCs-exo and miR-214-3p/CD151 should be further validated in cell experiments. Moreover, other mRNAs and potential signaling pathways targeted by miR-214-3p in AD processes deserve future investigations.

## Supplementary Material

Supplementary Table 1.docx
