# Supplementary material for: MicroRNA-214-3p Delivered by Bone Marrow Mesenchymal Stem Cells-Secreted Exosomes Affects Oxidative Stress in Alzheimer’s Disease Rats by Targeting CD151
Source: Organogenesis. 2025 Apr 27;21(1):2489673. doi: 10.1080/15476278.2025.2489673 (PMC12036478; doi:10.1080/15476278.2025.2489673)
Supplement: Supplementary Table 1.docx [file KOGG_A_2489673_SM6369.docx]

**Supplementary Table 1** Primer sequences for PCR

| Genes | Primers |
| --- | --- |
| microRNA-214-3p | Forward: 5'-ACAGCAGGCACAGACAGGCAG -3' |
|  | Reverse: 5'-GTGCAGGGTCCGAGGTATTC-3' |
| U6 | Forward: 5'-CTCGCTTCGGCAGCACA-3' |
|  | Reverse: 5'-AACGCTTCACGAATTTGCGT-3' |
| CD151 | Forward: 5'-ACTTCATCCTGCTCCTCATCAT -3' |
|  | Reverse: 5'-TCCGTGTTCAGCTGCTGGTA-3' |
| β-actin | Forward: 5'- CATCCGTAAAGACCTCTATGCCAAC -3' |
|  | Reverse: 5'-ATGGAGCCACCGATCCACA-3' |
